# Intraocular inflammation following aflibercept 8 mg: real-world data from a multicentre retrospective observational study

**DOI:** 10.1186/s40942-025-00768-0

**Published:** 2025-12-08

**Authors:** Lea-Noelle Stoehr, Philip Wakili, Warda Darwisch, Franziska Seufert, Robert P. Finger, Peter Szurman, Boris V. Stanzel

**Affiliations:** 1 Eye Clinic Sulzbach, Knappschaft Hospitals Saar, Knappschaftsklinikum Saar, An der Klinik 10, 66280 Sulzbach/Saar, Germany; 2 Klaus Heimann Eye Research Institute, Sulzbach/Saar, Germany; 3 Medical Faculty Mannheim, University Eye Clinic Mannheim, University of Heidelberg, Mannheim, Germany; 4 Curschmann Ophthalmology Practice, Ludwigshafen, Germany

**Keywords:** Aflibercept 8 mg, Intraocular inflammation, Adverse events, Real-world evidence

## Abstract

**Background:**

Real-world data on intraocular inflammation (IOI) following aflibercept 8 mg is limited. We report on a cluster of 12 eyes with aflibercept 8 mg-associated IOI detected at three centres between July 2024 and June 2025.

**Method:**

Primary outcome measures of the retrospective observational study were the classification of severity according to the SUN classification and the determination of the incidence of aflibercept 8 mg-associated IOIs. The inclusion criterion was on-label therapy in treatment-naive and patients switched to aflibercept 8 mg who developed sterile inflammation within 1 to 14 days after injection without a history of uveitis. Data was collected by means of medical history, clinical biomicroscopic examination, optical coherence tomography and fluorescein angiography. The incidence was calculated based on the number of injections performed at the three centres between July 2024 and March 2025.

**Results:**

The case series comprises 12 eyes from 10 patients (7 women, 3 men) with post-injection IOI after an average of 1.9 days (SD: 0.6); nAMD (80.0%) / DME (20.0%). IOI occurred on average after 2.3 injections (SD: 1.6), with 91.7% pretreated without IOI event (including aflibercept 2 mg (66.7%)). Anterior uveitis developed in 16.7%, intermediate uveitis in 66.7% and posterior uveitis in 16.7%. The incidence was 1.4% per aflibercept 8 mg injection (11 eyes out of 775 injections; 95% CI: 0.6–2.2%).

**Conclusion:**

Initial real-world data indicate a slightly increased risk of IOIs with aflibercept 8 mg compared to the pivotal studies, some of which are fulminant and difficult to distinguish from endophthalmitis.

## Background

The approval of new anti-vascular endothelial growth factor (VEGF) agents offers more optimised treatment options for neovascular and exudative retinal diseases. Since early 2024, aflibercept 8 mg was approved in the EMA region (Eylea HD^®^, Bayer/Regeneron), with a safety profile comparable to that of aflibercept 2 mg (Eylea^®^, Bayer/Regeneron), as demonstrated in the approval studies [1, 2].

Complications such as endophthalmitis, intraocular inflammation (IOI), intraocular hemorrhage, or retinal detachment are rare in the context of intravitreal anti-VEGF therapy. Among these, IOI has gained increasing relevance, particularly with the introduction of newer agents. Each anti-VEGF drug appears to carry a specific risk profile in this regard. In a retrospective study by Williams et al., the risk of IOI under real-world conditions was 0.16% for aflibercept 2 mg, compared with 0.1% for bevacizumab (Avastin^®^, Roche/Genentech) and 0.02% for ranibizumab (Lucentis^®^, Novartis/Genentech). More recently, higher rates of IOI have been reported for brolucizumab (Beovu^®^, Novartis), which has raised particular attention regarding drug-specific safety profiles [3]. The risk of Faricimab (Vabysmo^®^, Roche/Genentech) -associated IOI in the approval studies is 0.6–1.3%, depending on the indication [4, 5], which was also confirmed by initial real-world data with an incidence of 0.86% per eye [6]. Abicipar Pegol, the first *Designed Ankyrin repeat protein* (DARPin), was not approved by the Food and Drug Administration (FDA) in 2020 due to the disproportionately high incidence of IOIs of 15% per treated patient reported in the phase 3 studies (CEDAR, SEQUOIA) and a cluster of retinal vasculitides at 1.8%. Although no further cases of vasculitis were reported after a modification of the manufacturing process, the risk of IOI remained significantly higher at 8.9% compared to classic anti-VEGF preparations (MAPLE study) [7, 8].

The severity of an IOI can range from a subclinical course to potentially vision-threatening inflammation comparable to endophthalmitis, which typically occurs 5 days after injection [9]. This must be distinguished from immune-mediated vasculitis, which was first observed in connection with brolucizumab (Beovu^®^, Novartis) and whose frequent occurrence was only detected after approval [10]. As real-world data on the incidence and clinical presentation of IOIs after aflibercept 8 mg are currently limited, increased vigilance regarding the occurrence of adverse side effects should also be exercised here initially. We report on a cluster of 12 eyes with an aflibercept 8 mg-associated IOI, which were detected at three centres between July 2024 and June 2025.

## Method

### Study overview

This is a retrospective observational study comprising a data analysis of patients with aflibercept 8 mg-associated IOI at three centres. These were a tertiary care facility (A: Eye Clinic Sulzbach) and two practices (C, D). Individual follow-up examinations were also carried out outside the three institutions, including at the University Eye Clinic in Mannheim, some of whose data was also submitted. The data was collected as part of a comprehensive quality control programme from the routine clinical care of patients with neovascular AMD (nAMD) and Diabetic macular edema (DME) at the Eye Clinic Sulzbach, which has been in practice since 2022. The study is limited to data collected between July 2024 and June 2025.

### Patient selection

Only adult patients (minimum age 18 years) who received injections of 8 mg aflibercept for nAMD or DME were included. These included both treatment-naive patients who had not previously received any other intravitreal injections and patients who had switched to aflibercept 8 mg with other anti-VEGF preparations or intravitreal steroids in the past. The inclusion criterion was the occurrence of sterile inflammation within 1 to 14 days after injection. The patients were classified as having anterior, intermediate or posterior uveitis based on the SUN classification (standardisation of uveitis nomenclature) [11]. Anterior chamber inflammation was evaluated using a 1 × 1 mm slit beam and graded based on the number of inflammatory cells from 0 to 4+ [12]. Vitreous inflammation was assessed according to the SUN vitreous haze scale ranging from 0 to 4+ (0 = no haze, 0.5 + = slight blurring of the optic disc margin, 1 + = disc and vessels visible but mildly blurred, 2 + = moderate blurring with the disc still visible, 3 + = disc barely visible, and 4 + = disc not visible) [13]. For this study, clinically significant IOI was defined as the presence of anterior chamber cells ≥ 0.5 + and/or vitreous haze ≥ 1+, or the occurrence of retinal vasculitis, retinitis, or optic nerve inflammation detected on fundus examination or multimodal imaging.

Patients with (exogenous) endophthalmitis post injectionem, in whom a pathogen was detected in an anterior chamber or vitreous sample, were excluded from the analysis. In cases with clinical suspicion of an infectious aetiology, anterior chamber or vitreous taps were performed for microbiological testing, including PCR analysis. The diagnosis of IOI was established when no pathogen was detected and the clinical course was consistent with sterile inflammation rather than infection. In particular, a prompt improvement under corticosteroid therapy was considered a key diagnostic indicator. A known history of anterior, intermediate or posterior uveitis, as well as the presence of silicone oil bubbles after intravitreal injection, was considered an exclusion criterion in order to minimize potential confounding.

### Data collection

Data were collected through medical history and a standardized ophthalmological examination including corrected distance visual acuity (CDVA), intraocular pressure measurement, biomicroscopic examination with a slit lamp, and SD-OCT (Spectralis(R) Heidelberg Engineering, Heidelberg, Germany). If retinal involvement was suspected, fundus photography and fluorescein angiography (FLA) were also performed. All injections were conducted under strictly aseptic conditions in a room specialised for intravitreal injections. Prior to injection, all patients underwent periocular disinfection with 0.5% povidone-iodine solution, followed by multiple applications of 1% povidone-iodine to the conjunctival sac with an appropriate exposure time. In accordance with the recommendations of the DOG (German Ophthalmological Society) and BVA (Professional Association of Ophthalmologists in Germany), no antibiotics were administered post-injection [14]. A routine follow-up examination of the patients was performed within 4 days after the injection.

The calculation of the incidence of aflibercept 8 mg-associated IOIs is based on the number of injections performed at centres A, B and C between July 2024 and March 2025. One patient who received the injection outside the facilities was excluded from the calculation. As detailed patient-level data were only available from centre A, patient-based incidence could be determined exclusively for this site. To ensure methodological consistency and comparability across all centres, incidence was therefore calculated per injection for the overall cohort.

### Evaluation parameters

The primary evaluation parameter was the determination of the severity of inflammation based on the distinction between anterior, intermediate and posterior uveitis, as well as the collection of data on the incidence of Aflibercept 8 mg-associated IOIs.

Secondary parameters included an exploratory analysis of the clinical presentation, covering symptoms at initial presentation, the number of aflibercept 8 mg injections administered prior to the event, time of occurrence, clinical findings (anterior chamber reaction, vitritis, retinal involvement), therapeutic measures, and the course of visual acuity.

### Statistical analyses

Data analysis was performed using standard descriptive statistics. Incidence rates were expressed as the arithmetic mean of the observed event rate with 95% confidence intervals. For small sample sizes, the Clopper–Pearson method was applied. All calculations were performed using Microsoft Excel (Microsoft Corp., Redmond, WA, USA).

## Results

The case series comprises a total of 12 eyes from 10 patients (7 women, 3 men, average age: 81.2 years) who developed aflibercept 8 mg-associated IOI after an average of 1.9 days (SD: 0.6, within 1–3 days) post-injection, see Table 1. The therapy was performed in the context of nAMD (80.0%) or DME (20.0%). The IOI occurred on average after 2.3 injections (SD: 1.6) of aflibercept 8 mg, with the majority of patients (91.7%) having previously received anti-VEGF preparations, including aflibercept 2 mg (66.7%), without developing sterile inflammation. In 9 of 12 eyes, the syringe used could be determined retrospectively. In 9 eyes, the medication was prepared from a vial. In 8 eyes, the batch KT0LT75 was administered.

**Fig. 1:**
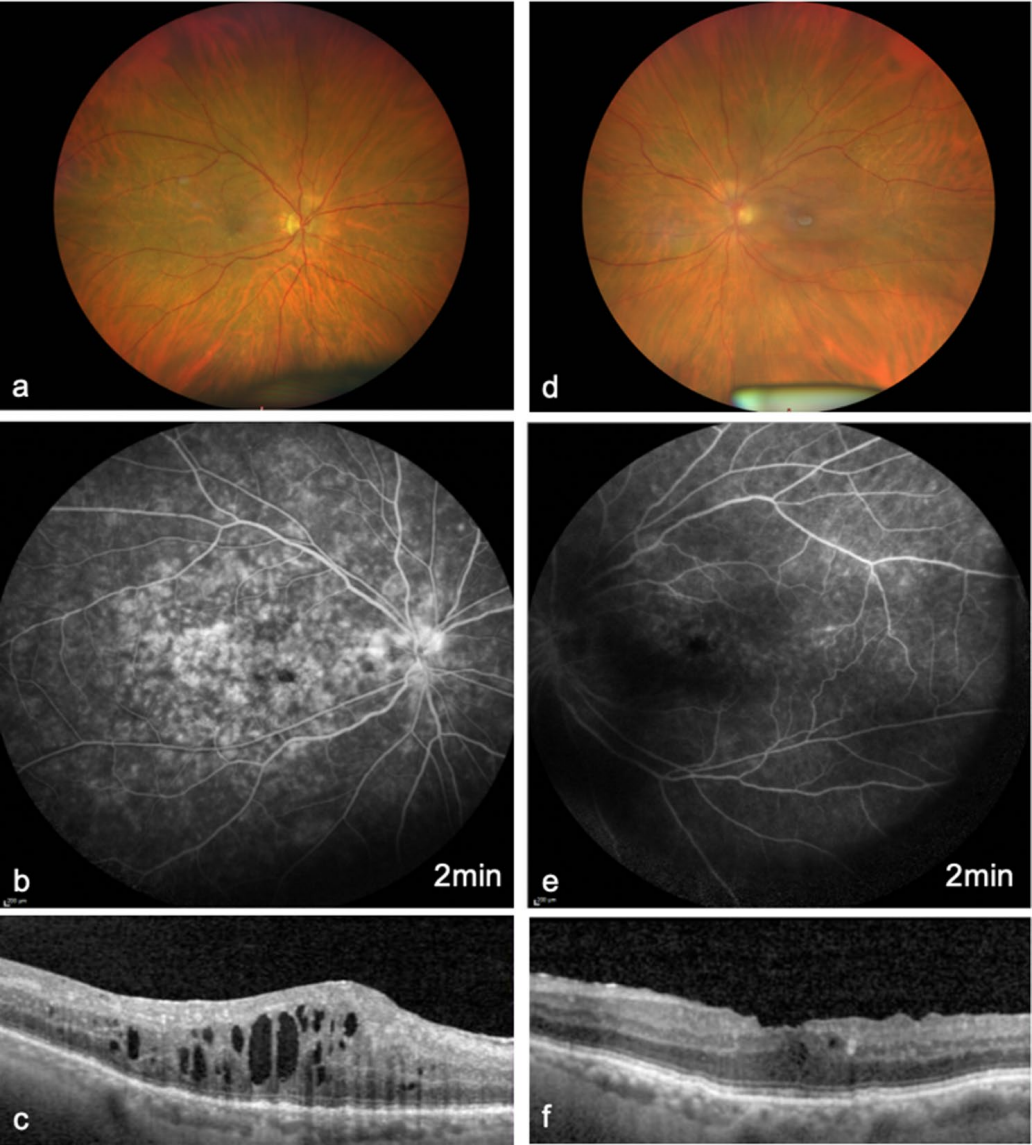
Fundus photograph (**a, d**), FLA (**b, e**) and OCT imaging (**c, f**) of a patient with anterior uveitis in the right eye (**a, b, c**) and intermediate uveitis in the left eye (**d, e, f**) with blurred view of the retina due to vitreous cells and snowballs. No systemic evaluation for a potential infectious or systemic cause of the uveitis was conducted; therefore, a coincidental non-infectious uveitis cannot be entirely excluded

**Fig. 2:**
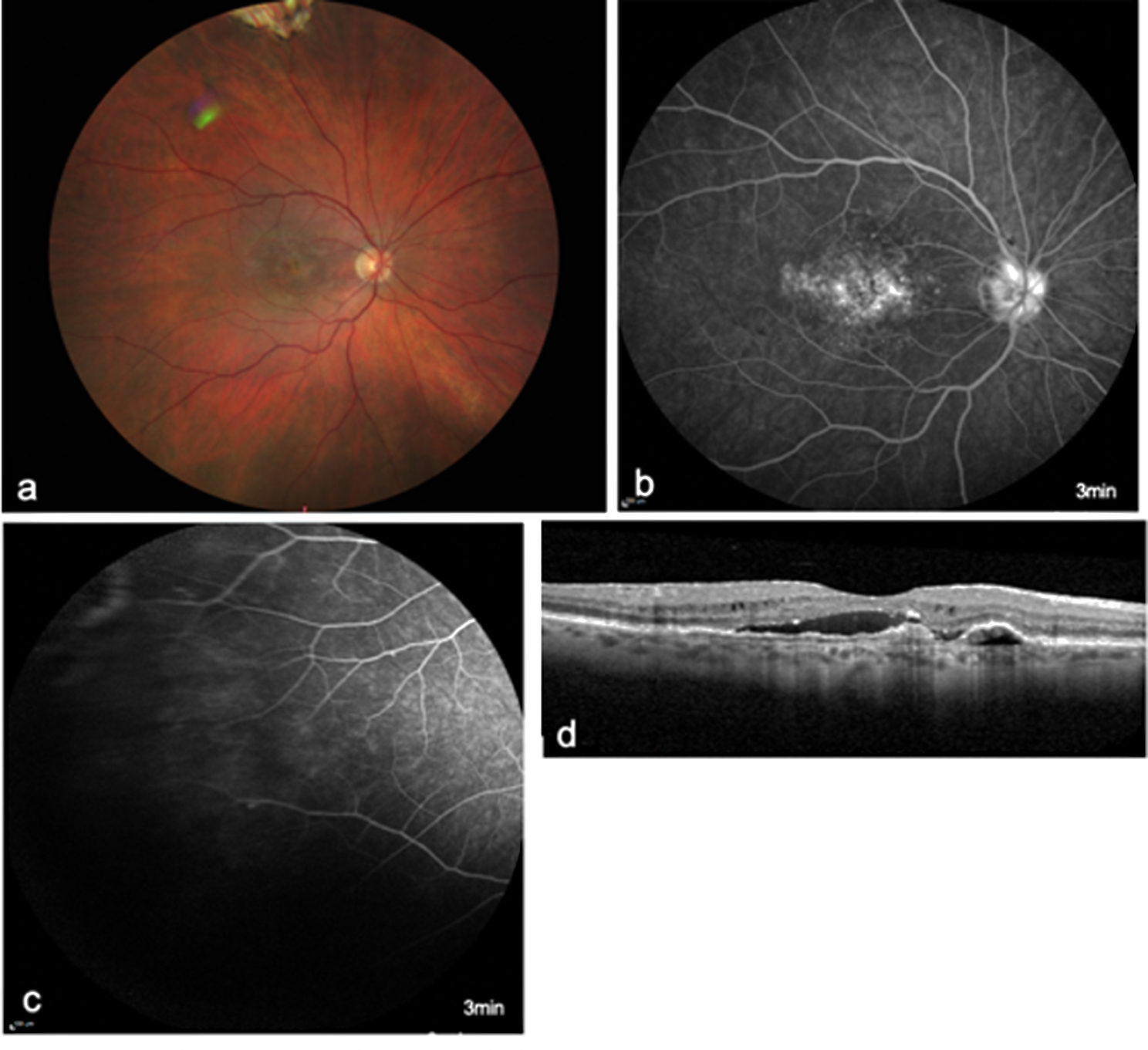
Fundus photograph (**a**), FLA (**b, c**) and OCT imaging (**d**) of a patient with posterior uveitis in the form of hyperfluorescence of the optic disc (**b**) and a subtle barrier disruption of the peripheral vessels (**c**)

**Fig. 3:**
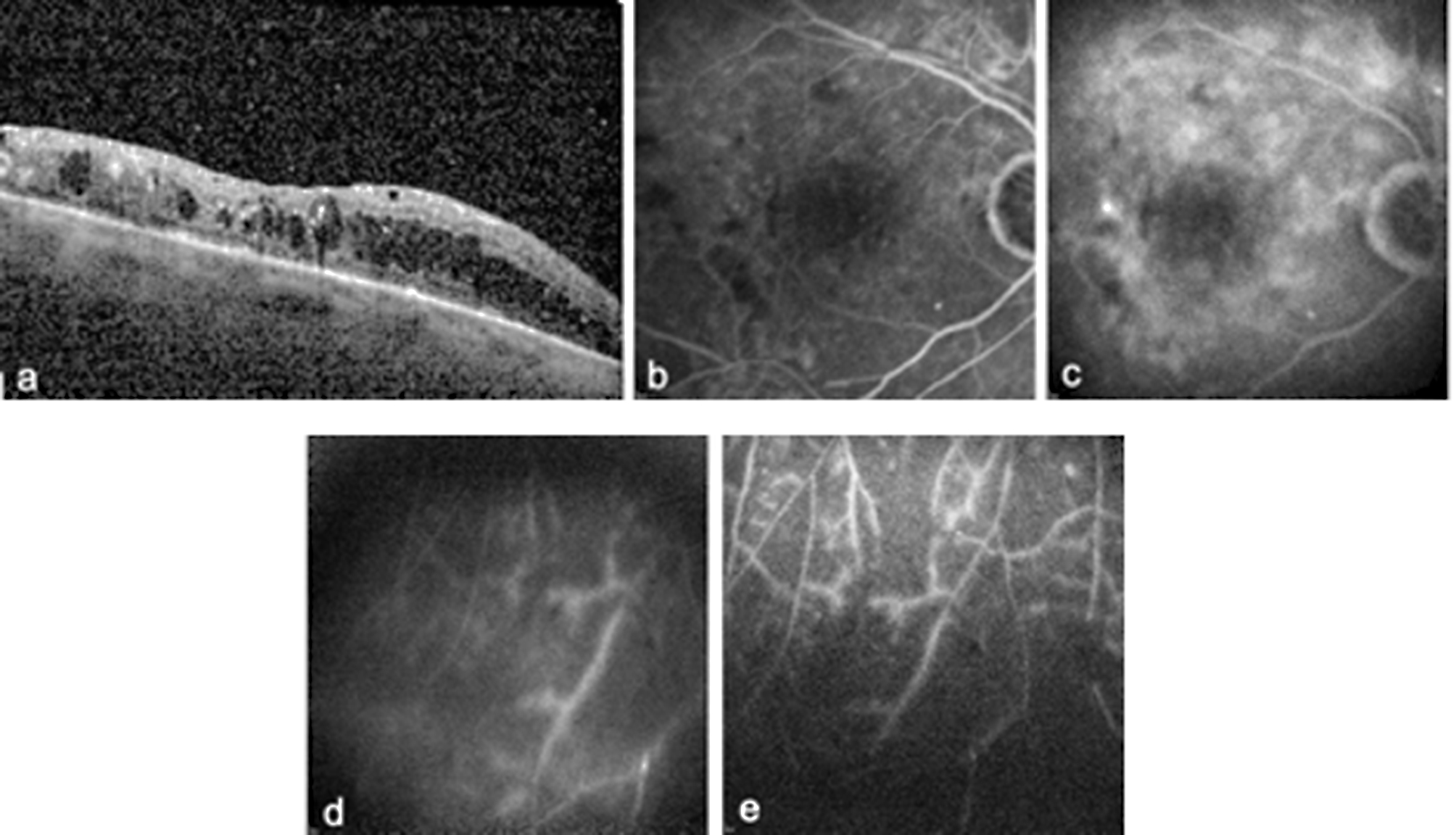
OCT (**a**) and FLA imaging (**b, c, d, e**) of a patient with severe non-proliferative diabetic retinopathy after vitrectomy with occlusive vasculitis of the peripheral vessels with fine vascular occlusions, leaks and spot haemorrhages in the periphery (**d, e**)

The most common symptom was subjective visual acuity reduction (83.3%), followed by pain (41.7%) and photophobia (25.0%). 16.7% of eyes developed anterior uveitis, 66.7% developed intermediate uveitis and 16.7% developed posterior uveitis (Figs. 1, 2 and 3). Clinical examination revealed anterior chamber cells in 66.7% of the eyes, endothelial cell precipitates in 50.0% and vitreous cells in 66.7%. Two patients also showed retinal involvement. One patient exhibited optic disc hyperfluorescence accompanied by barrier dysfunction of the peripheral retinal vessels (Fig. 2), whereas the second patient demonstrated occlusive vasculitis characterized by peripheral vascular occlusions with vascular leakage and scattered peripheral hemorrhages (Fig. 3).

Treatment consisted of topical corticosteroid administration in 75.0% of eyes and antibiotic eye drops in 33.3%. In addition, 25.0% of patients received parabulbar injections of triamcinolone acetonide. Furthermore, systemic antibiotics were administered in 20.0% of cases, and 10.0% of patients underwent intravenous therapy with Solu-Decortin H (250 mg). In 25.0% of eyes, exploratory pars plana vitrectomy was performed when endophthalmitis was initially suspected, but none of the vitreous samples yielded evidence of pathogens. 10.0% of the patients did not require further treatment. At presentation, 91.6% of eyes exhibited a reduction in visual acuity due to the IOI. The mean visual acuity loss at baseline corresponded to 4.6 lines (logMAR, SD 3.7), with 50.0% showing an initial reduction of more than four lines. Complete remission of vision within 6 months was achieved in 63.6% of eyes. However, the exact time to full recovery could not be determined in all cases, as some follow-up examinations were conducted at longer intervals or at external centres. In the 36.3% of eyes with persistent visual acuity reduction, the mean loss remained 4.5 lines (SD 5.0). One case (Table 1, patient 5) progressed to total retinal detachment following exploratory vitrectomy. In another patient (Table 1, patient 7), visual acuity after resolution could not be assessed retrospectively because of loss to follow-up. In one patient (Table 1, patient 3), therapy with aflibercept 8 mg was continued after the IOI event, and no recurrence of IOI was observed. All other patients were switched to alternative anti-VEGF agents or to steroid preparations.

**Table 1:** Clinical characteristics of IOI after Aflibercept 8 mg

Patient Institution (A, B, C, D)	Age	Disease	Numbers of Injections of Aflibercept 8 mg before IOI	Days until Presentation	Visus before IOI	Visus at first Presentation of IOI	Visus after recovery	Symptoms	Anterior Chamber reaction	Vitreous reaction	Retinal Involvment	Type of Inflammation	Therapy
1 A	86	nAMD, PCOWG	3	-	0.1	0.08	0.1	Blurred Vision	Endothelial precipitates	-	-	Uveitis anterior	-
2 B	87	nAMD	1	1	0.1	0.025	0.1	Blurred Vision	-	Vitreous cells, Snowballs	-	Uveitis intermedia	Triamcinolon parabulbar, topical Steroids
3 B	82	nAMD	2	-	0.4	0.12	0.25	Blurred Vision	-	Vitreous cells	-	Uveitis intermedia	topical Steroids
4 A	86	R/L: DME	4	-	RA: 0.1 LA: 0.8	RA: 0.05 LA: 0.25	RA: 0.1 LA: 0.5	RA: - LA: Visual impairment	R/L: Anterior chamber cells, endothelial precipitates	RA: - LA: Vitreous cells, Snowballs	- -	RA: Uveitis anterior LA: Uveitis intermedia	RA: - LA: topical Steroids, 250 mg Soludecortin H iv
5 C	86	R/L: nAMD	1	2	RA: 0.9 LA: 0.9	RA: 0.6 LA: 0.1	RA: 1.0 LA: 0.05	R/L: Visual impairment RA: Pain	R/L: Anterior chamber cells, endothelial precipitates	R/L: Snowbanks		R/L: Uveitis intermedia	LA: Vitrectomy R/L: topical antibiotics and steroids
6 C	77	DME	1	2	0.4	Fingercount	0.25	Blurred Vision, Pain	Anterior chamber cells	Vitreous cells	Occlusive Vaskulitis of the peripheral vessels	Uveitis posterior	Vitrectomy, topical antibiotics, Triamcinolon parabulbar, systemic antibiotics
7 C	96	nAMD	1	2	0.3	0.2	-	Blurred Vision	Anterior chamber cells	Vitreous cells	-	Uveitis intermedia	Vitrectomy, topical antibiotics and steroids, systemic antibiotics
8 C	65	nAMD	6	1	0.25	0.1	0.25	Blurred Vision, Pain, Photophobia	Endothelial precipitates	Vitreous cells	Hot disc, barrier disorder of the peripheral vessels	Uveitis posterior	Triamcinolon parabulbar, topical Steroids
9 C	77	nAMD	2	2	0.5	0.5	0.5	Pain, Photophobia	Anterior chamber cells	Vitreous cells	-	Uveitis intermedia	topical Steroids
10 A	70	nAMD	2	3	0.1	0.025	0.1	Visual Impairment, Pain, Photophobia	Anterior chamber cells	Vitreous cells	-	Uveitis intermedia	topical Steroids

Figure  1 : Patient characteristics, clinical presentation, visual acuity course, ophthalmic findings, severity grading per SUN classification, and treatment of 12 eyes with aflibercept 8 mg-associated IOI across three centres (A: Eye Clinic Sulzbach; B, C: practices)

At the three centres a total of 775 intravitreal injections (centre A: 206, centre B: 165, centre C: 404) of aflibercept 8 mg were administered between July 2024 and March 2025, with 11 eyes (9 patients) developing an IOI. The frequency of aflibercept 8 mg-associated IOI in our study population was thus 1.4% per injection (11 eyes out of 775 injections; 95% CI: 0.6–2.2%). At centre A, a total of 68 patients were treated with aflibercept 8 mg, of whom 2 developed IOI. The incidence in this subgroup was therefore 2.9% per patient (2 out of 68 patients; 95% CI according to Clopper–Pearson: 0.4–10.3%).

## Discussion

The phase 3 registration trials PHOTON and PULSAR reported a comparable safety profile for aflibercept at both the 2 mg and 8 mg dosages, with an incidence of IOI of < 1% in PHOTON and 1% in PULSAR on a per-patient basis [1, 2]. The frequency of 1.4% IOI per injection (2.9% per patient at centre A) observed in our cohort is therefore not consistent with the pivotal trial results and it also suggests a elevated risk in real-world practice than previously assumed. However, with regard to the reported patient-based incidence, it should be noted that a potential selection bias may exist, as centre A represents a tertiary referral institution with a potentially more extensively pretreated patient population compared to B and C, which may have contributed to the higher incidence observed. There is currently another retrospective study by Binder et al. which shows a comparatively high incidence of 3.7% per injection (5 cases per 136 injections), suggesting a possible systematic underreporting of IOIs with mild or asymptomatic progression [15] .

Particular attention should be paid to the sometimes fulminant and early clinical presentation observed in our cluster, which may closely mimic infectious endophthalmitis. In this context, the timing of symptom onset can provide an important clue to the underlying etiology. While IOIs after aflibercept 8 mg occurred quite promptly after injection, with an average of 1.9 days, exogenous endophthalmitis is characterised by a slightly delayed onset within 3–7 days post-injection [10]. However, the sometimes marked anterior chamber and vitreous inflammation, accompanied by a substantial decrease in visual acuity, can make it challenging to differentiate this condition from early infectious endophthalmitis [16]. Overtreatment in the form of vitrectomy, with the associated follow-up procedures and risk of secondary complications, should thus be strictly avoided where clinical presentations and history permit.

Since approval, there have already been three further case series describing IOIs after aflibercept 8 mg. The case series by Hoffmann et al. reports a cluster of 8 IOIs after aflibercept 8 mg, which also showed vitritis with partial spillover into the anterior chamber without retinal involvement. All patients achieved complete remission promptly with topical steroid administration [17] .

The retrospective study by Binder et al. reported five cases of aflibercept 8 mg-associated IOI, which, in contrast to our cohort, were characterized by a mild course with only transient visual impairment in all patients. Treatment consisted of topical corticosteroids and, in some cases, additional oral steroids. Increased vigilance is warranted regarding potential retinal involvement. In our series, two patients presented with posterior uveitis, ranging from peripheral occlusive vasculitis to optic disc hyperfluorescence accompanied by barrier dysfunction of the peripheral retinal vessels. In this context, it should be noted that the patient’s non-proliferative diabetic retinopathy could present with similar angiographic findings. However, the presence of anterior chamber and vitreous cells, together with the improvement following corticosteroid therapy, supports an aflibercept 8 mg–associated pathogenesis. Moreover, fluorescein angiography performed one year prior to the event showed no comparable retinal changes. Nevertheless, progression of microangiopathic alterations related to diabetic retinopathy cannot be entirely ruled out. In the pivotal aflibercept 8 mg registration trials, only a single case of post-injection chorioretinitis was documented, while none of the patients treated with aflibercept 2 mg developed posterior uveitis [1, 2]. It should also be considered that fluorescein angiography in our study was performed only in cases with suspected retinal involvement. Therefore, systematic underreporting of posterior involvement cannot be excluded. Further studies with routine use of fluorescein angiography in aflibercept 8 mg–associated IOI would be required to better approximate the true incidence of retinal involvement in real-world settings.

The study by Matsumoto et al. also points to an increased risk of aflibercept 8 mg-associated vasculitis. This includes a retrospective evaluation of 34 patients of Japanese origin, three of whom developed IOI after aflibercept 8 mg. Clinically, all three patients showed sterile inflammation in the form of retinal vasculitis with segmental vascular involvement, which was asymptomatic in all patients [18] .

The pathogenesis of IOI associated with VEGF inhibitors is considered multifactorial. In principle, patient-related factors, drug-related mechanisms, and procedural aspects of the injection process have all been discussed in the literature. In the latter case, particular attention has been directed to the potential formation of protein aggregates related to silicone oil-coated injection needles, as well as to possible contamination arising from vials disinfected with alcohol without allowing sufficient drying time [19, 20]. Drug contamination or improper storage can also lead to the formation of proteins with immunogenic effects [16]. In this context, contamination limited to a single batch is unlikely, as the batch we recorded was KT0LT75, while the batch used in the publication by Hoffmann et al. was KT0LKCK.

Whereas the literature primarily attributes brolucizumab-associated vasculitis to an immune complex reaction mediated by anti-drug antibody (ADA) formation, this mechanism is likely to play only a minor role in IOI following aflibercept 8 mg [21]. The risk of ADA formation after aflibercept 2 mg in the VIEW studies is only 1–3%, whereas ADA formation was detected in 36–52% of cases with brolucizumab [22]. Moreover, the majority of patients in our series, as well as those reported by Binder et al., Hoffmann et al., and Matsumoto et al., had previously been treated with aflibercept 2 mg without developing inflammation. IOI occurred only after switching to the higher 8 mg dosage. Matsumoto et al. attributed the increased incidence of aflibercept 8 mg–associated IOI to the fourfold stronger inhibition of VEGF-A achieved with the 8 mg formulation compared to the 2 mg dosage [23]. Stronger VEGF blockade may also result in damage to healthy endothelial cells. This facilitates increased migration of proinflammatory cells, such as monocytes, into the injured tissue, thereby contributing to narrowing of the vascular lumen. In addition, monocytes promote enhanced release of proinflammatory cytokines, which reduces endothelial barrier function and can manifest as retinal vascular leakage on FLA [24]. In its most severe form, this can lead to vasculitis with an occlusive component [18] .

The relatively small number of IOIs cases limits the generalizability of the results, even though a trend in clinical manifestation is emerging in view of comparable results from previously published cases. Further multicentre studies and ongoing monitoring are needed to determine the actual incidence of aflibercept 8 mg-associated IOIs in real world. Conclusions regarding potential risk factors cannot be drawn due to the limited sample size and the lack of information on systemic diseases or immunological predispositions; these aspects should be addressed in future studies. Furthermore, the retrospective nature of data collection and the irregularity of follow-up visits—some of which were conducted at external centres—may have resulted in gaps in documentation. The relatively short observational period precluded the evaluation of long-term outcomes and potential delayed-onset inflammatory events. Another limitation of this study is the absence of a control group, which makes it difficult to definitively attribute the observed IOI cases solely to aflibercept 8 mg. Without a comparator group receiving other anti-VEGF agents during the same period, potential confounding factors such as underlying ocular comorbidities, prior treatment history, or systemic inflammatory predispositions cannot be fully excluded.

In addition, the potential occurrence of culture-negative infections that could have been misclassified as IOI represents another limitation of this study. To minimize this risk, only cases with a clinical presentation and disease course clearly consistent with IOI were included, particularly those showing a rapid response to corticosteroid therapy.

In summary, initial real-world data indicate an increased risk of IOIs with aflibercept 8 mg compared to the approval studies. Particular attention should be paid to the sometimes-fulminant initial clinical presentation, which complicates differentiation from infectious endophthalmitis, as well as to the potential occurrence of retinal involvement. This underscores the importance of comprehensive awareness of aflibercept 8 mg-associated IOIs in this increasingly utilized anti-VEGF treatment.

## Data Availability

The datasets used and/or analysed during the current study are available from the corresponding author on reasonable request.

## Abbreviations

ADA: Anti-drug antibody formation
CDVA: Corrected distance visual acuity
DME: Diabetic macular edema
FLA: Fluorescein angiography
IOI: Intraocular inflammation
nAMD: neovascular AMD
OCT: Optical coherence tomography
PFS: Prefilled syringe
VEGF: Vascular endothelial growth factor
